# Phylodynamic analysis and spike protein mutations in porcine deltacoronavirus with a new variant introduction in Taiwan

**DOI:** 10.1093/ve/veab096

**Published:** 2021-11-24

**Authors:** Fu-Chun Hsueh, Cheng-Nan Wu, Marco Yung-Cheng Lin, Feng-Yang Hsu, Chuen-Fu Lin, Hui-Wen Chang, Jih-Hui Lin, Hsin-Fu Liu, Ming-Tang Chiou, Kuan Rong Chan, Chao-Nan Lin

**Affiliations:** Animal Disease Diagnostic Center, College of Veterinary Medicine, National Pingtung University of Scienceand Technology, Pingtung 912301, Taiwan; Department of Medical Laboratory Science and Biotechnology, Central Taiwan University of Science and Technology, Taichung 406053, Taiwan; Department of Medical Research, Mackay Memorial Hospital, Taipei 10449, Taiwan; Department of Nursing, Shu-Zen Junior College of Medicine and Management, Kaohsiung 821004, Taiwan; Department of Nursing, Yuh-Ing Junior College of Health Care and Management, Kaohsiung 80776, Taiwan; Animal Disease Diagnostic Center, College of Veterinary Medicine, National Pingtung University of Scienceand Technology, Pingtung 912301, Taiwan; Department of Veterinary Medicine, College of Veterinary Medicine, National Pingtung University of Science and Technology, Pingtung 912301, Taiwan; Animal Disease Diagnostic Center, College of Veterinary Medicine, National Pingtung University of Scienceand Technology, Pingtung 912301, Taiwan; Department of Veterinary Medicine, College of Veterinary Medicine, National Pingtung University of Science and Technology, Pingtung 912301, Taiwan; Graduate Institute of Molecular and Comparative Pathobiology, School of Veterinary Medicine, National Taiwan University, Taipei 10617, Taiwan; Department of Veterinary Medicine, School of Veterinary Medicine, National Taiwan University, Taipei 10617, Taiwan; Center for Diagnostics and Vaccine Development, Centers for Disease Control, Taipei 11561, Taiwan; Department of Medical Research, Mackay Memorial Hospital, Taipei 10449, Taiwan; Department of Bioscience and Biotechnology, National Taiwan Ocean University, Keelung 202301, Taiwan; Institute of Biomedical Sciences, MacKay Medical College, New Taipei City 25245, Taiwan; Animal Disease Diagnostic Center, College of Veterinary Medicine, National Pingtung University of Scienceand Technology, Pingtung 912301, Taiwan; Department of Veterinary Medicine, College of Veterinary Medicine, National Pingtung University of Science and Technology, Pingtung 912301, Taiwan; Program in Emerging Infectious Diseases, Duke-NUS Graduate Medical School, Singapore 169857, Singapore; Animal Disease Diagnostic Center, College of Veterinary Medicine, National Pingtung University of Scienceand Technology, Pingtung 912301, Taiwan; Department of Veterinary Medicine, College of Veterinary Medicine, National Pingtung University of Science and Technology, Pingtung 912301, Taiwan

**Keywords:** porcine deltacoronavirus, evolutionary rates, phylodynamics, phylogenetics, structural analysis

## Abstract

Porcine deltacoronavirus (PDCoV) is a highly transmissible intestinal pathogen that causes mild to severe clinical symptoms, such as anorexia, vomiting, and watery diarrhea in pigs. By comparing the genetic sequences of the spike glycoprotein between historical and current Taiwanese PDCoV strains, we identified a novel PDCoV variant that displaced the PDCoV responsible for the 2015 epidemic. This PDCoV variant belongs to a young population within the US lineage, and infected pigs carry high concentrations of the virus. It also has several critical point mutations and an amino acid insertion at position 52 that may enhance the affinity between the B-cell epitopes located in the N-terminal domain with its complementarity regions, consequently facilitating binding or penetration between the fusion peptide and cellular membrane. Furthermore, viral protein structure prediction demonstrated that these amino acid changes may change the ability of the virus to bind to the receptor, which may consequently alter virus infectivity. Our results hence suggest the emergence of new PDCoV strains in Taiwan with the potential for greater transmission and pathogenesis.

## 1. Introduction

Porcine deltacoronavirus (PDCoV) was first identified in Hong Kong in 2012 (Woo et al. 2012) and has caused severe economic loss to the swine industry worldwide recently (Jung, Hu, and Saif 2016). It is an enveloped virus with a large, capped and polyadenylated RNA genome of approximately 25,400 nucleotides (Hsueh et al. 2021). PDCoV belongs to the genus *Deltacoronavirus*, family *Coronaviridae,* and order *Nidovirales*. Other members of this genus are bird coronaviruses (CoVs), including bulbul CoV, thrush CoV, munia CoV, white-eye CoV, sparrow CoV, magpie robin CoV, night heron CoV, wigeon CoV, and common moorhen CoV (Woo et al. 2012). Birds are believed to be the original host for *Deltacoronavirus* (Woo et al. 2012). Indeed, genome sequence analysis indicates that PDCoV originated relatively recently from interspecies jumping between sparrow and pigs (Woo et al. 2012). Recent studies showed that PDCoV can infect human cells using host aminopeptidase N (APN) as a viral entry receptor (Li et al. 2018; Cruz-Pulido et al. 2021). In addition, PDCoV is also able to bind to the receptor in feline and chicken cells (Li et al. 2018; Liang et al. 2019). Therefore, this information also provides insight into potential zoonotic capabilities of PDCoV (Lednicky et al. 2021).

The spike (S) glycoprotein of coronaviruses is involved in the early steps of viral infection, promoting binding to host cell surface receptors for viral entry and mediating membrane fusion to facilitate viral infection (Zhou, Qiu, and Ge 2021). Mutations in the S gene contribute to the emergence of new CoV variants, and these mutations can be observed in both human CoVs and animal CoVs (Lin et al. 2021a). For example, severe acute respiratory syndrome coronavirus 2 (SARS-CoV-2) S glycoprotein D614G mutation has been demonstrated to increase entry efficiency and viral infectivity due to enhanced receptor-binding affinity (Li et al. 2020; Zhang et al. 2020; Ozono et al. 2021). The S glycoprotein is mainly composed of two subunits, S1 and S2. The S1 subunit serves as a receptor-binding unit and is further divided into an N-terminal domain (NTD) and receptor-binding domain (RBD) (Shang et al. 2018). RBD is the principal player in determining the host range of coronaviruses. For instance, RBD of SARS-CoV-2 binds strongly to human and bat angiotensin-converting enzyme 2 (ACE2), while PDCoV utilizes APN as an essential cellular receptor for viral entry (Li et al. 2018; Andersen et al. 2020). Recently, a few important mutations in the RBD-binding interface have been shown to be pivotal in binding of RBD to the virus receptors, suggesting that mutations in these residues could influence viral infectivity (Zhang et al. 2021). Similar to other CoVs, NTD of PDCoV can bind to the sugar moiety of mucin and exploit these sugars for viral entry (Shang et al. 2018; Xiong et al. 2018). Interestingly, enhancing anti-NTD antibodies of SARS-CoV-2 can also induce the open conformation of the RBD, by increasing the binding capacity of the S protein to ACE2 that consequently promote SARS-CoV-2 infection (Liu et al. 2021). Therefore, investigating how the NTD impacts infectivity of coronavirus warrants further investigation.

The S2 subunit induces fusion between the viral and the cellular membranes after binding to the host receptor. It is composed of the central helix N, fusion peptide (FP), heptad repeat region N, central helix C, heptad repeat region C, and transmembrane domain (Tang et al. 2020). The FP is highly conserved across the *Coronaviridae* family and is composed of mostly hydrophobic residues that inserts in the cellular membrane to trigger the fusion event (Sainz et al. 2005; Madu et al. 2009; Alsaadi, Neuman, and Jones 2019; Lai and Freed 2021). Of note, FP tends to be sensitive to point mutations, although mutations in most N-terminal residues have little or no effect on membrane fusion. Nonetheless, FP is critical for membrane fusion and the presence of a high degree of alpha-helix structure allows penetration into the outer leaflet of the target membrane with the kink at the phospholipid surface (Madu et al. 2009).

PDCoV is a highly transmissible intestinal agent, causing mild to severe clinical syndromes, such as anorexia, vomiting, and watery diarrhea in piglets and/ or sows (Jung, Hu, and Saif 2016). The clinical signs of PDCoV are similar to other swine enteric coronaviruses, such as transmissible gastroenteritis virus (TGEV), porcine epidemic diarrhea virus (PEDV), and swine acute diarrhea syndrome-coronavirus (Jung, Hu, and Saif 2016). Of note, the neonatal or suckling piglets are at higher risk of morbidity and mortality compared to the adult pigs. Although the mortality in piglets caused by PDCoV is comparatively lower than that caused by PEDV (Zhai et al. 2016; Zhang et al. 2019; Lin et al. 2021b), emerging studies reveal that discriminating the causative agents that causes mortality is critical for disease management, transmission, and infection control. PEDV has been considered as the dominant swine enteric coronavirus and caused huge economic losses in Taiwanese swine industry since late 2013 (Lin et al. 2014). Prior to early 2021, the prevalence of PDCoV infection was relatively low with only sporadic infection or co-infection with PEDV (Hsu et al. 2018; Lin et al. 2021b). However, starting in January, 2021, acute gastrointestinal syndromes such as anorexia, vomiting, and watery diarrhea in both piglets and sows occurred in the central Taiwan. A remarkable increase with similar clinical signs occurred in several pig herds from central to southern Taiwan. Most intestinal specimens were tested negative for TGEV and PEDV by real-time polymerase chain reaction (PCR) but positive for a high concentration of PDCoV, highlighting the potential for PDCoV to cause severe clinical manifestations in piglets and sows. Newly emerging PDCoV strains have been continually reported, but detailed information regarding the evolutionary history of PDCoVs in Taiwan is still cloaked.

Here, by examining the genetic sequences of PDCoV strains from 2015 and 2021, we identified a dominant variant of PDCoV in Taiwan that displaced the PDCoV that was detected in 2015. This variant exhibited higher virus loads in pigs with structural conformation changes in the S glycoprotein. The phylodynamic, evolutionary, and structural analysis on the complete S gene sequences are examined in this study.

## 2. Materials and methods

### 2.1 Specimen collection and history

During January, 2021, sows and/or suckling piglets had acute gastrointestinal signs such as anorexia, vomiting, and watery diarrhea in a 3300-sow farrow-to-wean pig farm located in central Taiwan. Rectal swabs collected from diarrheic sows and piglets were submitted to the Animal Disease Diagnostic Center, National Pingtung University of Science and Technology, Taiwan; subsequently, similar clinical signs were reported in succession in other pig herds from neighbor counties during late January to mid-March 2021. Intestinal specimens were quantified by quantitative reverse transcription PCR (RT-qPCR) as previously mentioned (Hsueh et al. 2021). The same protocol of RT-qPCR was performed for examining specimens collected from 2015 and 2021. The limit of detection for RT-qPCR was 1.68 genomic equivalents (GE)/µl. To examine the disparities of PDCoV viral loads between different years, we incorporated the results in 2015 (Lin et al. 2021b) in comparison to those detected in 2021. Viral loads were compared statistically using SAS 9.4 (Statistical Analysis System, SAS Institute Inc., Cary, NC, USA) with one-way analysis of variance and depicted using GraphPad Prism 6 (GraphPad Software, San Diego, CA, USA).

### 2.2 S gene sequencing and phylogenetic analysis

Total viral nucleic acids were extracted using the MagNA Pure LC total nucleic acid isolation kit (Roche Diagnostics, Mannheim, Germany); the complementary DNA (cDNA) was synthesized utilizing the PrimeScript^TM^ RT reagent kit (Takara Bio Inc., Kusatsu, Shiga, Japan). For sequencing of the full-length S gene, the cDNA was completely amplified by PCR with reagents of KAPA HiFi HotStart ReadyMix (Roche) and five specific primer pairs (Supplementary Table S1). Multiple sequence alignment of various PDCoV strains was established using Clustal W in the Molecular Evolutionary Genetics Analysis (MEGA), version 7 software program, in order to construct the phylogenetic trees by methods of neighbor-joining (NJ) in the Kimura 2-parameter model.

### 2.3 Phylodynamic analysis

The aforementioned multiple sequence alignment of PDCoV sequences was further performed for phylodynamic analysis. The significant temporal signal of the dataset was verified by TempEst software for molecular clock analysis (Rambaut et al. 2016). The evolution rates of PDCoV were estimated by the Bayesian Markov chain Monte Carlo (MCMC) method provided with BEAST software v1.10.4 (Suchard et al. 2018). The SRD06 as the best-fit nucleotide substitution model was examined for coding regions under Bayesian analyses to pursue higher resolution (Shapiro, Rambaut, and Drummond 2006). In terms of the demographic model, the evolutionary and population dynamics under strict and relaxed molecular clock models were estimated and taken the constant size, expansion growth, and GMRF Bayesian Skyride into consideration. The best-fit demographic model and clock model were, therefore, assessed by marginal likelihood with path sampling and stepping-stone sampling (Baele et al. 2013). The convergence of MCMC chains was achieved and further yielded the effective sample size above 200 (Lanfear, Hua, and Warren 2016). The final maximum clade credibility (MCC) was constructed by Tree Annotator v.1.8.4 with a burn-in period of 10 per cent and edited by Figtree v.1.4.2 (http://tree.bio.ed.ac.uk/).

### 2.4 Selection pressure of S glycoprotein genes

The foregoing phylogenetic results were performed to determine selection pressures on the S glycoprotein of PDCoV. The ratio of nonsynonymous substitutions (dN) and synonymous substitutions (dS) per site based on the maximum likelihood tree in the appropriate substitution model has been calculated using the single likelihood ancestor counting (SLAC) with fixed effects likelihood (FEL) methods at a significance level of 0.05 (Kosakovsky Pond and Frost 2005). The Bayesian test for selection acting on individual sites was conducted using Fast Unconstrained Bayesian AppRoximation (FUBAR), with the posterior probabilities set at 0.95 (Murrell et al. 2013). All methods were implemented in the HyPhy package and accessed through the Datamonkey web-server interface (http://www.datamonkey.org) (Weaver et al. 2018).

### 2.5 Three-dimensional structural analysis of S glycoprotein variations and secondary structure prediction

The amino acid sequences of the S glycoprotein of the PDCoV strain 110-504 and reference strain 104-553 were respectively analyzed by the program BLAST based on the default parameters. Serial viral strains were randomly selected from the aligned results, which were equipped with similar identities above 98.5 per cent, and displayed as the target of subsequent multiple sequence analysis. Multisequence alignments were established through the Clustal W algorithm. To better understand the capability of infectivity-enhancing sites on coronaviruses, we added the protein sequence of SARS-CoV2 as an additional reference strain (Liu et al. 2021), mutated the corresponding SARS-CoV-2 S glycoprotein sequence, and finally constructed structural homology modeling through the Swiss-model server (https://swissmodel.expasy.org/). The template, SARS-CoV2 with Fab fragment of enhancing antibodies PDB: 7DZX, was adopted in this study and demonstrated the structural function on Open-Source PyMol (https://github.com/schrodinger/pymol-open-source) for further analysis. The fusion peptide sequences of PDCoV variants were submitted to the RaptorX Property Web server and analyzed with default parameters (Wang et al. 2016). The eight-state protein secondary structure element was defined by DSSP (hydrogen bond estimation algorithm).

## 3. Results

### 3.1 Clinical and histological findings of PDCoV

**Figure 1.**
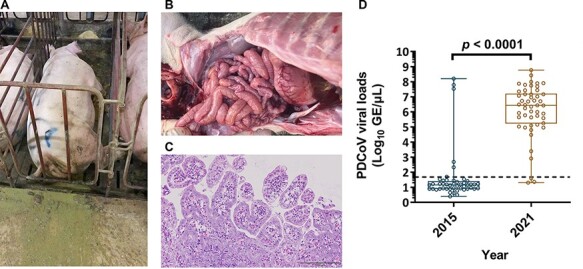
The naturally infectious status of PDCoV in the pig farm. A. An example of one PDCoV-infected sow, which manifested lethargy and severe watery diarrhea. B. Gross morphology of small intestine from a PDCoV-confirmed piglet. C. Microscopic lesions on the tissue section of jejunum from a PDCoV naturally infected piglet. D. Mean levels of PDCoV viral loads were detected by RT-qPCR between 2015 and 2021.The mean values of PDCoV GE were log_10_ transformed and presented as GE/µl. The limit of detection for RT-qPCR was 1.68 log_10_ GE/µl marked as the dotted line. PDCoV viral loads in 2015 and 2021 were as follows, 1.57 ± 1.69 and 6.09 ± 1.69 log_10_ GE/µl, respectively. Error bars indicated standard deviations.

Clinically, all sows manifested loss sprite, anorexia, and severe watery diarrhea (Fig. 1A), while suckling piglets that exhibited severe watery diarrhea also experienced dehydration with milk curd vomitus. After necropsy, the small intestines were found to be diffusely reddish and gas-distended with accumulation of yellowish contents (Fig. 1B). Histopathological examination revealed the presence of lesions that were composed of locally extensive to diffused villus atrophy and fusion, multifocally vacuolated, and degenerated villous enterocytes in those diarrheal piglets (Fig. 1C). Moreover, numerous lymphocytes and eosinophils infiltrated the lamina propria and in some instances, the blood vessels. Using real-time PCR, all samples were found to be PEDV RNA-negative and PDCoV RNA-positive. Of note, similar clinical features were successively reported in other pig herds from neighboring counties during late January to mid-March 2021, amounting to a total of 50 clinical samples collected from 13 pig herds, all of which were submitted to the Animal Disease Diagnostic Center, National Pingtung University of Science and Technology, Taiwan. Among these cases, only one was co-infected with PEDV, highlighting that PDCoV was the causative agent for the observed severe disease manifestations. The titers of PDCoV viral loads in intestinal and/or fecal specimens are depicted in the Fig. 1D, with a mean viral load of 6.09 ± 1.69 log_10_ GE/µl. Comparing the viral loads of PDCoV in this study to the viral loads measured during the 2015 epidemic (1.57 ± 1.69 log_10_ GE/µl), we observed a ∼10^5^ log increase in viral loads (*P* < 0.0001). These findings hint at the possibility of an outbreak of a novel PDCoV variant that is potentially more virulent in Taiwan.

### 3.2 Sequencing, phylogenetic, recombination, and amino acid analysis of PDCoV

**Figure 2.**
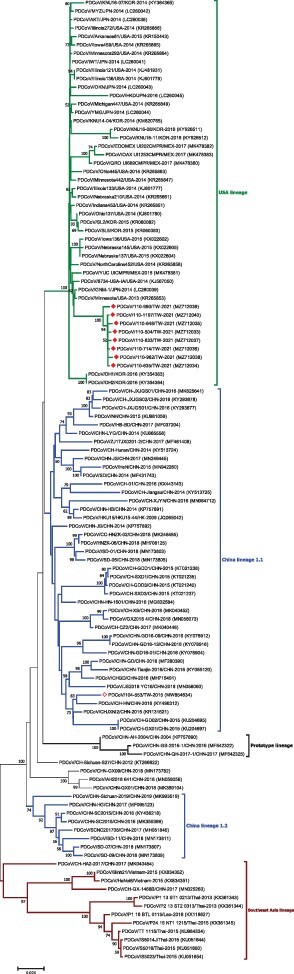
Phylogenetic analysis based on the full-length spike gene sequences of PDCoV strains. Circular phylogenetic trees were constructed by the NJ method, using the Kimura 2-parameter (K2P) model. The red solid diamond represents the PDCoV strain in 2021; the hollow solid diamond indicates the Taiwanese historical strain in 2015. The scale bar shows nucleotide substitutions per site.

As genetic mutations in the S glycoprotein have been previously demonstrated to impact viral virulence, we sequenced the S genes collected from a total of eight PDCoV strains that were collected from different pig farms in three distinct sites within Taiwan. As expected, the complete S gene sequences were 3,483 bp in length for all eight strains (GenBank accession no. MZ712033–MZ712040). The nucleotide sequences of all strains in the present study showed 99.76 per cent to 99.94 per cent identity to one other. Based on previous classification criteria (Hsueh et al. 2021), our sequenced PDCoV strains exhibited the highest nucleotide identity (98.28 per cent to 99.24 per cent) to the US lineage (length 3,483 bp), 97.10 per cent to 98.43 per cent nucleotide identity to the Chinese lineage (estimated length 3,480 or 3,483 bp), 97.77 per cent to 98.07 per cent nucleotide identity to the prototype (early China) lineage (length 3,480 or 3,483 bp), with the lowest nucleotide identity (95.21 per cent to 96.11 per cent) to the Southeast Asia lineage (length 3,480 or 3,483 bp) (Fig. 2). Interestingly, as compared to the historical Taiwan PDCoV 104-553 variant with the length of 3,480 bp, our PDCoV strains collected in 2021 showed only 97.64 per cent to 97.71 per cent nucleotide similarity, supporting the emergence of new PDCoV variants circulating in Taiwan currently (Fig. 2). Multiple alignments of amino acids of the full-length S glycoprotein deduced that Taiwanese PDCoV variants in 2021 could be an adolescent posterity evolved from the viral strains of the US lineage. In comparison with other PDCoVs, Taiwanese PDCoV strains in 2021 were featured with scattered and diverse amino acid mutations (0.5 per cent to 3.5 per cent range), including A^14^V, L^38^P, S^40^R, K^96^R, H^123^Y, D^134^N, V^137^A, and V^200^G mutations in NTD of the S1 subunit, and V^302^L, V^326^I, and T^337^S mutations in RBD of the S1 subunit, and A^698^S along with S^700^P mutation in the FP of the S2 subunit. In addition, one specific insertion at ^52^N was identified in these PDCoV strains.

### 3.3 Evolutionary rates and phylodynamics of PDCoV

**Figure 3.**
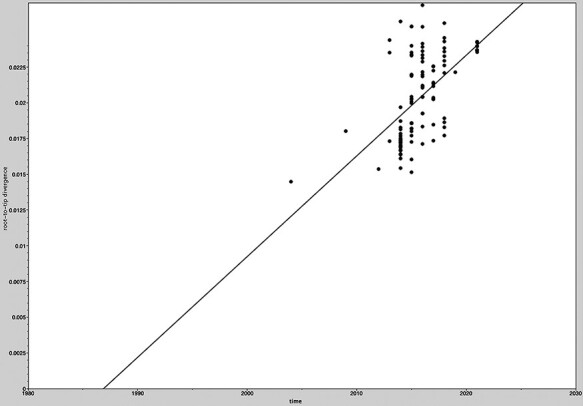
Root-to-tip regression analyses of the phylogenetic temporal signal in the PDCoV dataset. Plots of the root-to-tip genetic distance against sampling time shown for neighbor-joining phylogeny estimated from alignment 111 complete spike glycoprotein gene sequences, sampled between 2004 and 2021.

**Figure 4.**
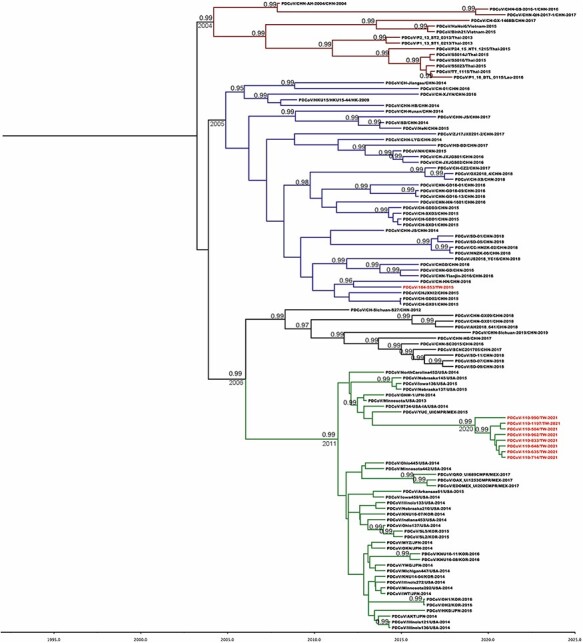
MCC tree of PDCoV inferred from 111 complete spike glycoprotein gene sequences. The MCC tree was constructed with 10 per cent burn-in by Tree Annotator v 1.8 implemented in the BEAST software package. Red text represents the Taiwanese PDCoV isolates in this study. Numbers beside the branches are posterior probability values and branch time. Only posterior probability values above 0.95 are shown.

All Taiwanese PDCoV sequences along with the global and historical reference strains were included to generate the phylodynamic reconstruction. First of all, we investigated the temporal signal and quality through the TempEst software. The root-to-tip regression analysis showed a positive correlation between the collection year and genetic divergences (Fig. 3), supporting that the dataset is suitable for molecular clock analysis. The uncorrelated lognormal-relaxed clock model and GMRF Bayesian Skyride population model were determined as the best-fit models for BEAST analysis. The estimated nucleotide substitution rates of PDCoV S glycoprotein were 1.324 × 10^−3^ substitutions per site per year, with the 95 per cent highest posterior density (HPD) ranging from 1.07 × 10^−3^ to 1.577 × 10^−3^. The time to the most recent common ancestor (TMRCA) of PDCoV was estimated to be 2001 (95 per cent HPD: 1993–2003). The evolutionary rate of the synonymous positions was determined at 1.987, which was significantly higher than the nonsynonymous positions (0.507) in the S glycoprotein region (Table 1). The MCC tree further manifested that all Taiwanese PDCoVs except for the PDCoV/104-553/TW-2015 variant belonged to a single cluster and the divergence time started at approximately 2011 (Fig. 4). PDCoV/104-553/TW-2015 belongs to the prototype (early China) lineage, with divergence time being estimated at 2005. Interestingly, the cluster of current PDCoVs isolates was branched in 2020, suggesting that 2021 Taiwanese PDCoVs are a young population within the US lineage (Fig. 4). Using the SLAC, FEL methods with a *P*-value threshold of 0.05, and the FUBAR method with a posterior probability of 0.95, we ascertained several positive selection sites of PDCoV S glycoprotein protein, including the discovery of positive selection sites at amino acid positions 107 and 149. Additionally, positive selection sites at positions 44 and 698 were verified through FEL and the FUBAR methods. Residues 44, 107, 149, and 183 are located to the NTD within the S1 subunit, whereas site 698 is located in a helix with the fusion peptide in the S2 subunit. The mean nonsynonymous/synonymous (dN/dS) rate in the S glycoprotein was 0.277, indicating the presence of purifying selection (based on dN/dS < 1) (Table 2).

**Table 1.** Evolutionary rates for codon positions and TMRCA in the spike glycoprotein of PDCoV strains.

	TMRCA	Substitution rates per site per year (10^−3^)	Mean relative substitution rates	Standard error of mean
Spike glycoprotein		1.324 (1.07–1.577)^a^		
PDCoV	2001 (1993–2003)^a^			
1st + 2nd codon position			0.498 (0.449–0.549)^a^	3.039E–4
3rd codon position			1.987 (1.885–2.088)^a^	6.078E–4

a () indicated the lower and upper 95 per cent of HPD.

**Table 2.** Selection sites detected in the glycoprotein of PDCoV.

Positively selected site positions			Negatively selected site position			Mean *d*_N_/*d*_S_
SLAC^a^	FEL^a^	FUBAR^b^	SLAC	FEL	FUBA	
107, 149	44, 107, 123, 149, 183, 698	40, 44, 107, 149, 642, 698	93	143	138	0.277

a *P* value < 0.05;

b posterior probability ≥ 0.95.

### 3.4 Structural analysis of S glycoprotein variation reconstructed in the template of SARS-CoV-2

**Figure 5.**
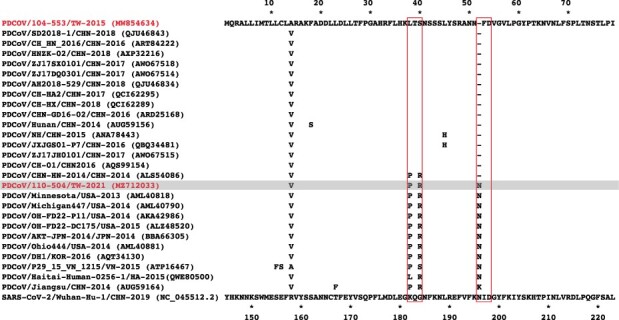
Multiple sequence alignment of coronavirus S proteins. The coronavirus strains included the spike protein of the Taiwanese PDCoV 104-553 and 110-504 strains (gray), reference historical PDCoVs, and SARS-CoV-2. Only those amino acid sequences differing from the Taiwanese PDCoV 104-553 are shown.

**Figure 6.**
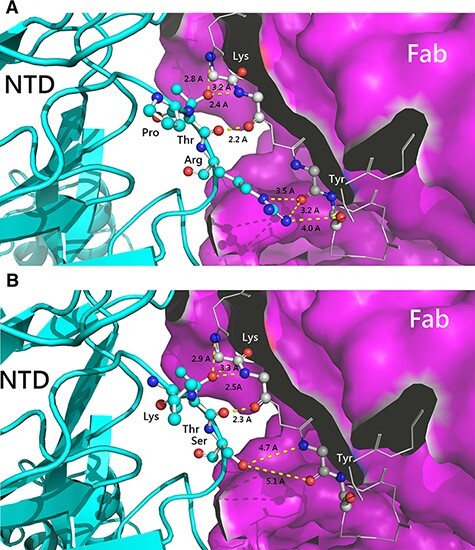
The combination capability of amino acid variation of the infectivity-enhancing site on the spike protein of SARS-CoV-2. Structural changes as a result of A. PTR epitope replacement of the 110-504 strain and B. LTS epitope of the 104-553 strain. NTD: N-terminal domain of spike protein and Fab: the antigen-binding fragment of infectivity-enhancing antibody.

The multiple sequence alignment of historical reference strains and the SARS-CoV-2 strain in different periods and regions was arranged, as shown in Fig. 5. As compared to the SARS-CoV-2 strain, all PDCoV variants exhibited a high degree of conservation in parts of specific amino acids, suggesting their importance in the stabilization of protein and functions. Although the amino acid sequences of different PDCoV strains showed high similarity between each other, unique amino acids mutations at the residues 38–40 were observed in the PDCoV/110-504/TW-2021 and PDCoV/104-553/TW-2015 strains, with amino acid sequences of Pro-Thr-Arg (PTR) and Lys-Thr-Ser (LTS), respectively. Interestingly, this region of PDCoVs coincidentally corresponded to the infectivity-enhancing site of SARS-CoV-2 (Liu et al. 2021), which could have accounted for differences in viral infectivity in the 2015 and 2021 virus strains. We thus replaced the reference SARS-CoV-2 S glycoprotein with the epitopes, PTR and LTS of Taiwanese PDCoV strains, to examine the effects of these amino acid changes on the virus structure. The linkage between ^182^Pro-^183^Thr on the NTD and ^58^Lys on the complementarity regions (CDRs) showed apparent differences. The length of the hydrogen bond to the infectivity-enhancing site was 2.2–3.2 A in the presence of the PTR epitope (Fig. 6A), whereas substitution to the LTS epitope increased the H-bond length to 2.3–3.3 A (Fig. 6B). This indicates that the binding ability of the PTR variant is similar to that of the LTS counterpart. However, as presence of the proline reduces the flexibility of the structure, changing the leucine amino acid residue may affect the binding of the epitope to the antibody. The substitution of two mutated amino acids, ^184^Ser and ^184^Arg, on the NTD also exhibited disparate structural conformational changes when they combined to the ^60^Tyr on the CDRs. Substituting the ^184^Ser to ^184^Arg increased the capability of the formation of H-bonds (Fig. 6A), which significantly enhanced the affinity between this epitope and the CDRs.

**Figure 7.**
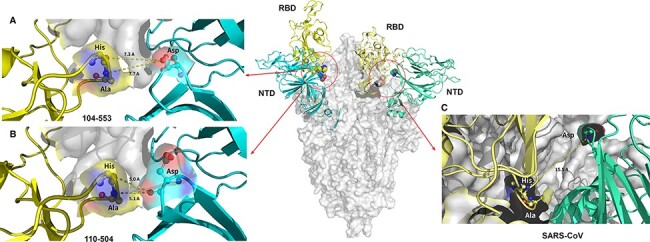
Conformational change of SARS-CoV-2 spike protein induced by the infectivity-enhancing antibody-binding site to calculate the structural protein stability. Structural conformation of proteins with A. the substituted residue, ^52^-(deletion) of the PDCoV 104-553 strain. B. The substituted residue, ^52^N, of the PDCoV 110-504 strain. C. The native SARS-CoV-2 structure. RBD: receptor-binding domain; NTD: N-terminal domain.

The S glycoprotein is a homogenous protein tertiary structure with a polypeptide chain of the CDRs lying in the center area among three axes of symmetry linking to another polypeptide chain from the outer angle of the NTD (Fig. 7). When the infectivity-enhancing sites were combined with the NTD of the S glycoprotein, this could induce the protruding structure off the primeval platform. In the native structure, the residue ^517^His-^518^Ala originating from the A chain had no connection with the residue ^198^Asp derived from the B chain originating from RBDs (Fig. 7C). However, when the infectivity-enhancing sites contact with the NTD of the C chain, the B chain would reconfigure protruding from the primeval platform; meanwhile, the residue ^517^His-^518^Ala moved close to the residue ^198^Asp forming a weak bond. The amino acid at ^198^Asp of SARS-CoV-2 is highly conserved with that at ^54^Asp of PDCoV (Fig. 5). In our study, the position of residue at ^52^-/N (Asn) of different pathogenic PDCoVs is positioned relatively close to the residue ^54^D (Asp). Therefore, we used the template of SARS-CoV-2 to simulate two amino acid sequences ^50^NN-FD^55^ of the 104-553 strain and ^50^NNNFD^55^ of the 110-504 strain, in order to figure out if the protein structure would be reconstructed (Fig. 7A, B). The results demonstrated that the binding capability between the reserved ^198^Asp of the NTD and ^517^HA-^518^Ala of RBDs derived from another polypeptide chain was significantly altered. In the PDCoV/110-504/TW-2021 strain, this insertion could provide better binding ability, which may facilitate the structural stability of RBDs after protein structure rearrangement.

### 3.5 Structural analysis of fusion peptides in PDCoV

**Figure 8.**
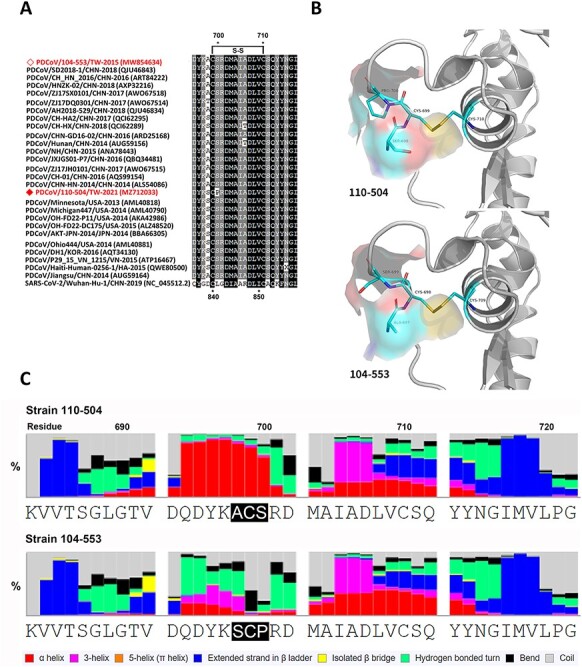
Comparison of fusion peptides of PDCoV 110-504 and 104-553 strains. A. Multiple sequence alignment of Taiwanese PDCoVs 110-504 and 104-553 strains and reference historical PDCoVs. B. The structural simulation of the fusion peptide surface near the polymorphic sites. Due to residue ^52^- (deletion) of the PDCoV 104-553, ^697^ACS^699^ corresponds to ^698^SCP^700^ of PDCoV 110-504. C. The secondary structure prediction was determined by eight-class analyses.

The multiple sequence alignment of Taiwanese PDCoV strains and historical reference strains manifested a specific amino acid mutation (A^698^S) (Fig. 8A). Precisely, in the PDCoV 110-504 strain, another amino acid mutation (S^700^P) was also discovered, which indirectly triggers the formation of the disulfide bond between the ^699^Cys and ^710^Cys (Fig. 8B). The residue ^698^ACS^700^ and ^698^SCP^700^ in the fusion peptide were located in the junction between the S1 and S2 subunits when PDCoV is not binding to the target receptor. Thus, the amino acid mutation (A^698^S) could induce a slight variation of molecular surface charges (Fig. 8C), which can influence the combination capability of glycoprotein. In addition, the protein secondary structure prediction demonstrated that this mutated residue ^697^ACS^699^ to ^698^SCP^700^ was ascribed to residue ^52^N deletion of PDCoV 104-553. This alteration could affect the formation of the hydrogen bond with other proteins or the combination capability with the lipid bilayer. As compared to the PDCoV/104-553/TW-2015 strain, the PDCoV 110-504 variant was relatively prone to form the alpha helix, which made this viral strain become more hydrophobic to provide stronger stability in the process of entering the lipid bilayer (Fig. 8C).

## 4. Discussion

PDCoV has become a massive concern to the swine industry and global health since coronaviruses have been shown to surmount interspecific barriers and generate viral variants among different animal hosts (Woo et al. 2012). Examples include PDCoV spread from wild birds to pigs (Boley et al. 2020; Ye et al. 2020) and the pandemic SARS-CoV-2 from bats to humans (Zhou et al. 2020; Wacharapluesadee et al. 2021). In this study, we demonstrated an outbreak of PDCoV that is associated with the US lineage-originated viruses and disseminated throughout the south-central areas of Taiwan around the spring of 2021. Acute gastrointestinal signs in piglets and sows in most of the clinical cases in 2015 were relatively mild except for the single case 104-553; however, the same intestinal signs in almost all cases in 2021 were instead severe. Interestingly, the virus sequence in 2021 is more distantly related to the genotype of PDCoV that previously occurred in 2015 (Hsueh et al. 2021). The rate of substitution of the current Taiwanese PDCoVs were estimated in our results to be 1.324 × 10^−3^ substitutions per site per year, was similar to that observation of US lineage by He et al. (2020) (1.21 × 10^−3^ substitutions per site per year), reaffirming that the US lineage displaced the local Taiwan strains found in 2015. Currently, the PDCoV epidemic is merely noted in 2021 in Taiwan. Based on phylodynamic analyses, the evolutionary origin of two PDCoV genotypes in Taiwan appeared to be alienated, indicating variable disseminated routes, but the exact time point and approach of the introduction of this US lineage-originated PDCoV remains unknown. One possibility is that this virus might invade Taiwanese swine population in 2020 through imported breeding pigs. Another possibility is the potential transmission of current PDCoV from birds to pigs in Taiwan.

The phylogeny of PDCoV can be preliminarily classified into four phenotypes: the prototype (early China), the China, the USA, and the Southeast strains (Hsueh et al. 2021). Previous challenge studies have shown that Chinese or US lineage-originated PDCoV could result in high titers of viral shedding and severely clinical syndromes (Suzuki et al. 2018; Zhang et al. 2019). Moreover, the prevalence of co-infection between PDCoV and other porcine coronaviruses may synergistically exacerbate disease severity in pigs (Ajayi et al. 2018). In Taiwan, the PDCoV epidemic in 2015 was dominated by the Chinese lineage viral strains with comparatively lower viral shedding (Hsueh et al. 2021); in contrast, the 2021 strains dominated by the USA-based variants manifested significantly soaring viral loads in fecal specimens. Among emerging porcine coronaviruses, PEDV and PDCoV have dominated the epidemic trends since 2014 (Wang et al. 2019; Zhang et al. 2019). Previous studies showed that the infectious ratios of PEDV were higher than those of PDCoV or co-infection by PEDV and PDCoV (Zhai et al. 2016; Zhang et al. 2019; Lin et al. 2021b). Indeed, co-infection of PEDV is detected during the 2015 Taiwan epidemic. Interestingly, we show that PDCoV infection, without PEDV co-infection, can also cause severe pathological behaviors in pigs. Our data hence encourage more comprehensive research on the interactions between PEDV and PDCoV strains.

Numerous amino acid mutations have reported the ability of coronaviruses to mutate between and within hosts (Perez-Rivera et al. 2019; Hsueh et al. 2020). Of note, point mutations located in the highly specific N-glycosylation can have drastic effects on transmission, survival, and recognition between viruses and hosts (Vigerust and Shepherd 2007). The eccentric mutation at position 134 (Asp-to-Asn [D^134^N]) within NTD, situated in the highly specific N-glycosylation site, was recorded in the PDCoV/110-962/TW-2021 strain, which has first been reported in PDCoVs. The S glycoprotein of CoV plays key roles in binding to host cell receptors for viral entry and eliciting neutralizing antibodies (Shang et al. 2018; Zhou, Qiu, and Ge 2021). Currently, our understanding about NTD of PDCoV is still limited. In the present study, our results indicated that mutations in the NTD of PDCoV can affect the protein structure in various ways: (1) capability of the formation of the hydrogen bond when the residue ^38^PTR^40^ is mutated to ^38^LTS^40^, thereby significantly enhancing the affinity between epitopes and CDRs (Fig. 6A); (2) the insertion of ^52^N could provide a better binding ability, which facilitated the structural stability of RBDs after the rearrangement of the protein structure. Moreover, residue 40 was found to be under positive selection, supporting that the ^38^PTR^40^ mutation to ^38^LTS^40^ may confer infection advantage of PDCoV.

The FP of the S2 subunit is composed of mostly hydrophobic residues that can interact with the cellular membrane to trigger fusion (Sainz et al. 2005; Madu et al. 2009; Alsaadi, Neuman, and Jones 2019; Lai and Freed 2021). Our results on FP also revealed that (1) the specific A^698^S mutation could induce a slight variation of molecular surface charges (Fig. 8C), which had high opportunities to influence the binding capability of S glycoprotein; (2) this mutated residue could affect the formation of hydrogen bonds with other proteins; and (3) the current PDCoV variant was relatively prone to construct the alpha helix, providing fortified stability during the entrance of the cellular lipid bilayer. Remarkably, S glycoprotein at residue 698 of PDCoV corresponds to residue 839 of SARS-CoV-2, which can alter viral infectivity (Li et al. 2020). Taken together, the current PDCoV variant shows significantly enhanced affinity between the epitope and the CDRs and can better facilitate the binding or penetration of the membrane to target cells, thereby augmenting both virus titers and their corresponding clinical signs as compared to the 2015 PDCoV strains. Although all above-mentioned amino acid mutations seem to have a strong connection with changed phenotypes, further experiments including challenge studies or comparison of growth kinetics will be needed to verify our discoveries.

Interspecific transmission in coronaviruses has received tremendous attention since the emergence of the delta variant of SARS-CoV-2 that has caused a worldwide pandemic. Previous studies have demonstrated that PDCoV has the capability of effectively infecting the cells of broad species, especially humans and chickens (Li et al. 2018; Liang et al. 2019; Cruz-Pulido et al. 2021). Hence, the avian species could be an available route of transmission and was even considered to be intermediate hosts for PDCoV (Boley et al. 2020). Indeed, the susceptibility of chickens to PDCoV infection further supports this notion (Liang et al. 2019). All the evidence hints that the interspecific transmission of PDCoV is potentially feasible, although the detailed mechanism remains unclear so far.

## 5. Conclusion

In summary, we identified a novel PDCoV variant in Taiwan in 2021, which is a population within the US lineage. We further reveal that the PDCoV variant has several critical point mutations within the S glycoprotein, theoretically changing the affinity between the epitope and the CDRs and assisting in binding or penetration into the membrane of target cells that consequently promotes viral infection. Our finding thus provides insights into the plausible mechanisms underlying increased transmission and pathogenesis in the novel PDCoV variant emerging in Taiwan, with the potential to spread to other parts of the world if the transmission is not properly managed.

## Supplementary Material

veab096_Supp

## Data Availability

PDCoV genomes have been submitted to the GenBank under the following accession number: MZ712033–MZ712040.
