# Supplementary material for: Phylodynamic analysis and spike protein mutations in porcine deltacoronavirus with a new variant introduction in Taiwan
Source: Virus Evol. 2021 Nov 24;7(2):veab096. doi: 10.1093/ve/veab096 (PMC8634457; doi:10.1093/ve/veab096)
Supplement: veab096_Supp [file veab096_supp.zip › Development of a UPL probe-based real-time PCR assay of PDCoV in Taiwan.pdf]

Taiwan Veterinary Journal, Vol. 47, No. 1 (2021)
DOI: 10.1142/S1682648521500013

World Scientific
www.worldscientific.com

Taiwan Vet J Downloaded from www.worldscientific.com
by Fong Kiaw Wong on 11/01/21. Re-use and distribution is strictly not permitted, except for Open Access articles.

DEVELOPMENT OF A UPL PROBE-BASED REAL-TIME PCR ASSAY OF PORCINE DELTACORONAVIRUS IN TAIWAN

Chao-Nan Lin[*,†,¶], Ming-Tang Chiou[*,†,¶], Fu-Chun Hsueh[†], Wei-Hao Lin[*,†], Chuen-Fu Lin[*,†], Cheng-Yao Yang[‡] and Shara Png Si Wei[§]

[*]*Department of Veterinary Medicine*
College of Veterinary Medicine
National Pingtung University of Science and Technology
Pingtung 91201, Taiwan

[†]*Animal Disease Diagnostic Center*
College of Veterinary Medicine
National Pingtung University of
Science and Technology
Pingtung 91201, Taiwan

[‡]*Graduate Institute of Veterinary Pathobiology*
College of Veterinary Medicine
National Chung Hsing University
Taichung, Taiwan

[§]*Diploma in Veterinary Technology*
School of Applied Science
Temasek Polytechnic 21
Tampines Avenue 1, Singapore

Received 17 March 2021
Accepted 14 May 2021
Published 28 July 2021

ABSTRACT

Porcine deltacoronavirus (PDCoV) causes clinical symptoms characterized by severe diarrhea and vomiting in neonatal piglets and pregnant sows, which is similar to those resulted from transmissible gastroenteritis coronavirus (TGEV) and porcine epidemic diarrhea virus (PEDV). Since PEDV was considered as the dominant enteric virus all round the world, PDCoV has been unwittingly overlooked due to its indistinguishable clinical signs with other porcine coronaviruses and relatively low death rates in the pig farm. Specimens which have been previously performed for the detection of PEDV in Animal Disease and Diagnostic Center, National Pingtung University of Science and Technology (NPUST) from January 5, 2015 to January 11, 2016 were examined by a novel universal probe library (UPL) probe-based

[¶]Corresponding authors: Chao-Nan Lin, Department of Veterinary Medicine, College of Veterinary Medicine, National Pingtung University of Science and Technology, Pingtung 91201, Taiwan. E-mail: cnlin6@mail.npust.edu.tw. Ming-Tang Chiou, Department of Veterinary Medicine, College of Veterinary Medicine, National Pingtung University of Science and Technology, Pingtung 91201, Taiwan. E-mail: mtchiou@mail.npust.edu.tw

real-time polymerase chain reaction (PCR). A total of 527 clinical specimens from pigs with diarrhea suspected were examined for PDCoV. Positive rates of PDCoV in small intestine and rectal swab were 4.3% (13/305) and 1.8% (4/222), respectively. Collectively, as to the total specimens, the detection rate is 3.2% (17/527). Our results provide development of a UPL probe-based real-time PCR assay and retrospective investigation of potentially circulating PDCoVs in the field in the whole 2015 and early 2016.

Keywords: Porcine deltacoronavirus; Porcine epidemic diarrhea virus; Co-infection; UPL probe.

Taiwan Vet J Downloaded from www.worldscientific.com by Fong Kiaw Wong on 11/01/21. Re-use and distribution is strictly not permitted, except for Open Access articles.

INTRODUCTION

Porcine deltacoronavirus (PDCoV), which was first discovered in Hong Kong in 2012 is an enveloped, positive sense, single-stranded RNA virus sized about 25.4 kb, belongs to the genus deltacoronavirus in the subfamily orthocoronavirinae of the family coronaviridae.[1] The PDCoV genome is composed of seven open reading frames (ORFs): four ORFs for structural proteins, including spike (S), envelope (E), membrane (M) and nucleocapsid (N), three ORFs for nonstructural, inclusive of ORF1a/b, nonstructural protein 6 (NS6) and nonstructural protein 7 (NS7).[2] The S protein plays a pivotal role in the function of receptor-binding ability, viral entrance and immunogenicity.[3] The M and N proteins are both relatively high conserved, and also determine the viral assembly and pathogenesis.[4]

PDCoV, also called porcine coronavirus HKU15 or swine deltacoronavirus (SDCoV), has been reported relating to diarrheal diseases in pigs in the US, Canada, South Korea, Japan, Thailand, China, Lao and Taiwan.[1,5–11] PDCoV causes similar clinical symptoms, characterized by severe watery diarrhea and dehydration, as transmissible gastroenteritis coronavirus (TGEV) and porcine epidemic diarrhea virus (PEDV) in neonatal and/or suckling pigs.[2,12,13] Even though the mortality in piglets caused by PDCoV is comparatively lower than that caused by PEDV, the differential diagnosis among these coronaviruses is still necessary to be developed because the various preventive or vaccination strategies can be performed in the field.[2,5] PEDV has caused huge economic losses, and further impaired the swine industry in Taiwan since late 2013.[3,14,15] Co-infection between PDCoV and PEDV had been previously reported in the USA and Canada; however, the prevalence and economic impacts of PDCoV remain vacuity in Taiwan during that period.[5,15] Thus, the aim of this study is to develop a Universal Probe Library (UPL)-based real-time polymerase chain reaction (PCR) assay for detecting PDCoV in Taiwan.

MATERIALS AND METHODS

Sample Collection

Small intestines and rectal swabs which were collected from 527 pigs (piglets and/or sows) were submitted to the Animal Disease and Diagnostic Center, National Pingtung University of Science and Technology for PDCoV detection from January 5, 2015 to January 11, 2016.

Construction of the Plasmid DNA Standard Curves

The PDCoV positive sample was cloned using the T&A cloning kit (Yeastern Biotech Co., Ltd., Taipei, Taiwan) and sequenced. PDCoV plasmids were extracted by a plasmid miniprep purification kit (GMbiolab Co., Ltd., Taichung, Taiwan) and quantified by measuring the OD260 using a NanoDrop 2000 (Thermo Fisher Scientific, Wilmington, DE, USA). A standard curve was depicted using 10-fold dilutions (10^8–10^2 copies number/μl) of the standard plasmid DNA.

UPL Probe-Based Real-Time PCR to Detect PDCoV

Nucleic acid extraction of clinical specimens was performed on a MagNA Pure LC 2.0 instrument by using the MagNA Pure LC total nucleic acid isolation kit (Roche Applied Science, Indianapolis, IN, USA). Following the cDNA synthesis was using PrimeScript™ RT reagent kits (Takara, Kyoto, Japan). The primer pair and probe, PDCoV-207F, PDCoV-207R and UPL probe 133, amplifying a 207-bp region based on a highly conserved region of three prime untranslated regions (3′-UTRs) were designed for detecting PDCoVs (Table 1). The real-time PCR were performed using the LightCycler Nano (Roche Diagnostics, Mannheim, Germany). Each 10 μL reaction mixture contained 5 μL of 2× Probes Master (Roche Diagnostics, Penzberg,

Table 1. Primer Pair and Probe Used in this Study.

Primer and Probe	Sequence (5′-3′)	Primer Length (bp)	Amplicon Size (bp)	Target	Position
PDCoV-207F	ATGCAAACTAGGGCTGGCTA	20	207	3′-UTR	25121-25140
PDCoV-207R	GGCGACTGCTACACCTACAAA	21			25327-25307
UPL probe #133	GGAGAAGG	8			25281-25288

Note: Nucleotide position is based on the porcine coronavirus HKU15 strain HKU15-155 (Accession No. JQ065043.2).

Table 2. Efficiency of the PDCoV UPL Probe-Based Real-Time PCR Assay.

Estimated PDCoV Plasmid DNA (Copies Number/μL)	Positive Results/ Tested Numbers	Mean $Cq\pm$ SD
10^8	10/10	14.64 ± 0.54
10^7	10/10	18.18 ± 0.39
10^6	10/10	21.82 ± 0.76
10^5	10/10	25.13 ± 0.89
10^4	10/10	28.60 ± 0.82
10^3	10/10	31.90 ± 0.76
10^2	10/10	35.60 ± 0.97

Note: The limit of detection for real-time PCR is $10^{1.68}$ copies number/μl.

Germany), 0.2 μM concentrations of PDCoV-207F and PDCoV-207R, 0.1 μM UPL probe #133 and 1 μL of the cDNA. The thermocycling conditions incorporated of 10 min at 95°C and 45 cycles of 15 s at 95°C, 15 s at 58°C and 15 s at 72°C.

Sensitivity, Specificity and Reproducibility Analysis

The sensitivity was evaluated via 10-fold serial dilutions of the standard plasmid DNA (10^8–10^2 copies number/μl), and tested for 10 times. The intra-(within-run) and inter-(between runs) assay reproducibility were evaluated via 10-fold serial dilutions of the standard plasmid DNA (10^8–10^2 copies number/μl), tested in triplicate on three different days. The specificity was assessed by testing nucleic acid extracts of the classical swine fever attenuated vaccine, PEDV, TGEV, porcine circovirus type 2, porcine parvovirus, porcine pseudorabies virus, *Escherichia coli* and *Clostridium perfringens*.

Statistical Analysis

The coefficient of variation (CV%) of the mean Cq values was calculated to assess the reproducibility. Fisher's exact test was used in the analysis of contingency tables and those groups with sample size larger than 10 which were analyzed. The P value < 0.05 was considered as significant difference and annotated with different letters.

RESULTS

UPL Probe-Based Real-Time PCR Amplification with Limit of Detection

Ten-fold serial dilutions of the PDCoV plasmid (10^8–10^2 copies number/μl) were used to construct the standard curve by scattered quantification values (Cq) in correspondence to the index values of copy numbers (Table 2). The standard curve was calculated by a linear range of six series of the concentration of the PDCoV plasmid in triplicates (Fig. 1). The slope and coefficient of determination (R^2) of regressive equation were -3.38 and 0.9922, respectively (Fig. 1). To figure out the limit of detection of the assay, $10^0 - 10^2$ copies number/μl of standard plasmids were tested additionally in 10 replicates. The limit of detection for real-time PCR is $10^{1.68}$ copies number/μl.

Reproducibility and Specificity of the UPL Probe-Based Real-Time PCR

The results of percentages of coefficient of variation (CV) of the mean Cq values in the intra-assay and inter-assay variability for the standard plasmid were distributed from 0.38% to 3.24%, and from 1.35% to 3.77% (Table 3). Meanwhile, other swine viruses and suckling pig diarrhea bacterial pathogens were examined to evaluate the specificity of the PDCoV probe-based real-time PCR. None of these samples were positive (data not shown).

Detection Rates in Small Intestines and Rectal Swabs from Pigs with PDCoV-Related Clinical Signs

A total of 527 clinical specimens from pigs with diarrhea suspected were examined for PDCoV. Positive rates of PDCoV in small intestine and rectal swab were 4.3% (13/305) and 1.8% (4/222), respectively (Table 4).

Taiwan Vet J Downloaded from www.worldscientific.com
by Fong Kiaw Wong on 11/01/21. Re-use and distribution is strictly not permitted, except for Open Access articles.

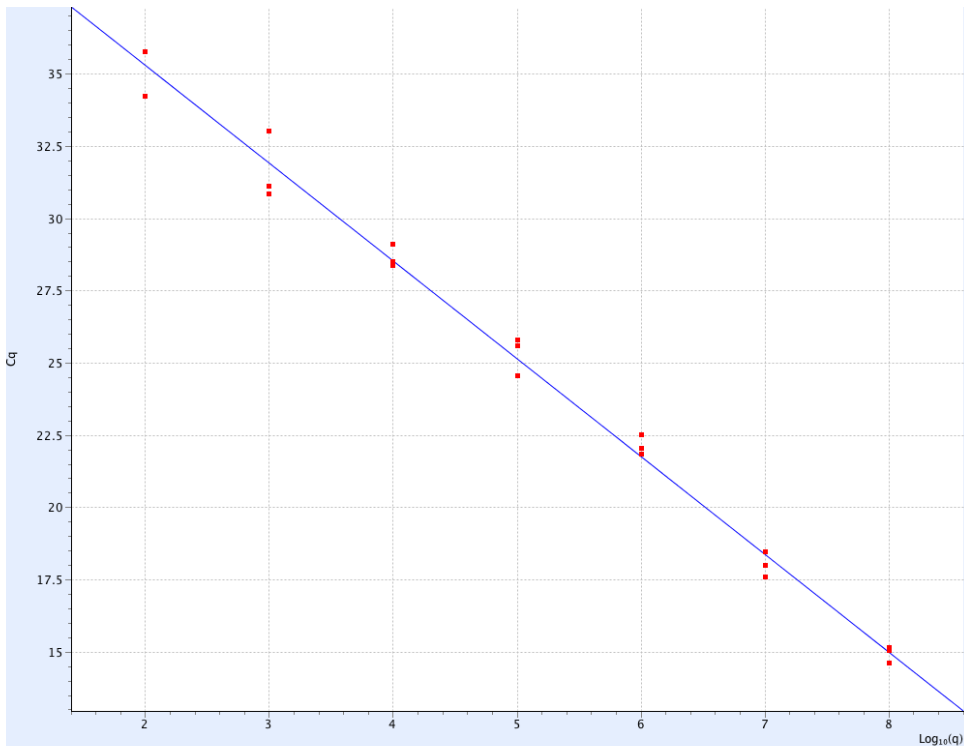

Fig. 1 Serial dilutions of PDCoV plasmids performed by UPL probe-based real-time PCR assay. A regression line among the scattered quantification values (*Cq*) with respect to logarithm values of the input copies number of the standard plasmid DNA was depicted.

Table 3. Reproducibility of the PDCoV UPL Probe-Based Real-Time PCR Assay.

Concentration of the PDCoV Plasmid (Copies Number/μl)	Intra-Assay Variability		Inter-Assay Variability	
	Mean *Cq*± SD	CV%	Mean *Cq*± SD	CV%
10^8	14.40 ± 0.35	2.43	14.90 ± 0.29	1.92
10^7	18.16 ± 0.46	2.58	18.00 ± 0.45	2.47
10^6	21.56 ± 0.70	3.24	22.12 ± 0.34	1.54
10^5	24.77 ± 0.51	2.07	25.29 ± 0.66	2.62
10^4	28.32 ± 0.11	0.38	28.64 ± 0.39	1.35
10^3	31.85 ± 0.55	1.71	31.64 ± 1.19	3.77
10^2	34.99 ± 0.16	0.46	35.23 ± 0.90	2.54

Note: The limit of detection for real-time PCR is $10^{1.68}$ copies number/μl.

Collectively, in terms of the 527 specimens, the detection rate is 3.2% (17/527) (Table 4).

DISCUSSION

PDCoV detection is performed by a probe-based real-time PCR targeting spike, membrane protein and replicase gene.[2,16–18] This study first develops a UPL probe-based real-time PCR for PDCoV detection. UPL includes 165 fluorescence-labeled DNA/Locked nucleic acid (LNA) hybrid hydrolysis probes, which detect 8 or 9 base pairs motifs. LNA is a ribonucleoside homolog with a 2′-O,4′-C-methylene linker or bridge and enables to allow short sequences of 8 to 9 bases to hybridize to complementary sequences with high affinity.[19] A single LNA base can increase the melting temperature about 9.6°C but the increase of melting temperature declines as more LNA bases.[20] The application of LNA to shorten the length of probes makes it more flexible to design probes and decreases the possibility of false negative results due to the high mutation rate of viruses. This assay is sensitive, specific and reliable with a reproducible limit of

Table 4. Positive Rates of PDCoV in Different Specimens.

Specimens	PDCoV
Small intestine	4.3% (13/305)
Rectal swab	1.8% (4/222)
Positive rate of all specimens	3.2% (17/527)

Note: Data were shown as percentage (positive number/examined number).

Taiwan Vet J Downloaded from www.worldscientific.com by Fong Kiaw Wong on 11/01/21. Re-use and distribution is strictly not permitted, except for Open Access articles.

Table 5. Positive Rates of PEDV and PDCoV of Different Specimen Types.

Specimen Type	PEDV Only	PDCoV Only	Co-Infection
Small intestine	47.5% (145/305)[a]	4.3% (13/305)[b]	3.6% (11/305)[b]
Rectal swab	45.5% (101/222)[a]	1.8% (4/222)[b]	3.2% (7/222)[b]
Positive rate of all specimens	46.7% (246/527)[a]	3.2% (17/527)[b]	3.4% (18/527)[b]

Note: Data were shown as percentage (positive number/examined number).

Taiwan Vet J Downloaded from www.worldscientific.com
by Fong Kiaw Wong on 11/01/21. Re-use and distribution is strictly not permitted, except for Open Access articles.

detection of $10^{1.68}$ copies number/μL of standard plasmid DNA for the purpose of targeting highly conserved 3′-UTR region by using a UPL probe.

The prevalence of PDCoV in this study was not as high as other coronaviruses, like PEDV during 2014–2015.[3,14] Due to the low detective rates of PDCoV, we include the molecular examination of PEDV, which had been well-developed in our previous research, to figure out if PEDV itself or co-infection between PDCoV and PEDV could show the different results. Based on Table 5, the detection rates of only PEDV infection are statistically significantly higher than those of only PDCoV infection and co-infection (p-value < 0.05). Surprisingly, the positive rates of co-infection in the rectal swab (3.2%, 7/222) and total specimens (3.4%, 18/527) slightly exceed those of only PDCoV infection in the same sample (1.8%, 4/222 and 3.2%, 17/527). A part of specimens was also performed for examining TGEV by real-time PCR, and the positive rate was only 4.9% (22/450) (data not shown). Those results showed the major pathogen causing viral diarrhea in Taiwan is PEDV, which is consistent with the previous research.[3] Previous study indicated that PDCoV existed in Taiwan since 2011.[9] The maternal antibody from infected sow herds might be a reason that makes the comparatively low prevalence of PDCoV. If the clinical signs or death rates are mild, pig farmers are usually not able to distinguish the PDCoV from *E. coli*, *C. perfringens*, TGEV, PEDV and rotaviruses. In addition, previous studies also suggested that the oral fluid has been used to monitor swine herds for PEDV and can also be a recommended specimen type for herd diagnosis for PDCoV, while the colostrum is not a recommended specimen type for diagnosis due to the lower positive rate.[21,22]

Suckling pigs were the major infective stage of both PDCoV and PEDV. They might have chances to transmit from sows to suckling pigs whenever the neutralizing antibody in the colostrum is not enough to protect suckling pigs. This could be related to sow herd immunity because the pathogenicity and virulence of PDCoV might not be as invasive as PEDV. Although the nursery period was not the major infective stage of PDCoV, the virus can still cause effects on the mortality, average daily gain and feed conversion ratio.[23] Nursery pigs can be the reservoir to increase the biosecurity risks in farms. As to the gilts and sows, their reproductive performance could be also be impaired due to PDCoV. They can further shed the viral particles to suckling pigs directly, which is similar to the infectious pattern of PEDV.[23]

CONCLUSIONS

PDCoV had been existing in the fields in Taiwan since 2015 and acted as a hidden pathogen causing the economic losses. Although the detection rates of PDCoV in different specimens were relatively low, the economic losses caused by PDCoV should still be regarded. In this study, we provide the development of a UPL probe-based real-time PCR assay and the information of potentially circulating PDCoVs in the field in the whole 2015 and early 2016. Further investigations on the surveillance of outbreak and phylogenetic analysis of PDCoV are necessary.

CONFLICT OF INTEREST

The authors declare that they have no competing interests.

ETHICAL APPROVAL

The study did not involve any animal experiment. The Institutional Animal Care and Use Committee (IACUC) of National Pingtung University of Science and Technology did not deem it necessary for this research group to obtain formal approval to conduct this study.

REFERENCES

1. Woo PC, Lau SK, Lam CS, Lau CC, Tsang AK, Lau JH, Bai R, Teng JL, Tsang CC, Wang M, Zheng BJ, Chan KH, Yuen KY, Discovery of seven novel Mammalian and

avian coronaviruses in the genus deltacoronavirus supports bat coronaviruses as the gene source of alphacoronavirus and betacoronavirus and avian coronaviruses as the gene source of gammacoronavirus and deltacoronavirus, *J Virol* **86**:3995–4008, 2012.
2. Wang L, Byrum B, Zhang Y, Detection and genetic characterization of deltacoronavirus in pigs, Ohio, USA, 2014, *Emerg Infect Dis* **20**:1227–1230, 2014.
3. Hsueh FC, Lin CN, Chiou HY, Chia MY, Chiou MT, Haga T, Kao CF, Chang YC, Chang CY, Jeng CR, Chang HW, Updated phylogenetic analysis of the spike gene and identification of a novel recombinant porcine epidemic diarrhoea virus strain in Taiwan, *Transbound Emerg Dis* **67**:417–430, 2020.
4. Zhang H, Liang Q, Li B, Cui X, Wei X, Ding Q, Wang Y, Hu H, Prevalence, phylogenetic and evolutionary analysis of porcine deltacoronavirus in Henan province, China, *Prev Vet Med* **166**:8–15, 2019.
5. Ajayi T, Dara R, Misener M, Pasma T, Moser L, Poljak Z, Herd-level prevalence and incidence of porcine epidemic diarrhoea virus (PEDV) and porcine deltacoronavirus (PDCoV) in swine herds in Ontario, Canada, *Transbound Emerg Dis* **65**:1197–1207, 2018.
6. Lee S, Lee C, Complete genome characterization of Korean porcine deltacoronavirus strain KOR/KNU14-04/2014, *Genome Announc* **2**, 2014.
7. Suzuki T, Shibahara T, Imai N, Yamamoto T, Ohashi S, Genetic characterization and pathogenicity of Japanese porcine deltacoronavirus, *Infect Genet Evol* **61**:176–182, 2018.
8. Janetanakit T, Lumyai M, Bunpapong N, Boonyapisitsopa S, Chaiyawong S, Nonthabenjawan N, Kesdaengsakonwut S, Amonsin A, Porcine deltacoronavirus, Thailand, 2015, *Emerg Infect Dis* **22**:757–759, 2016.
9. Dong N, Fang L, Zeng S, Sun Q, Chen H, Xiao S, Porcine deltacoronavirus in Mainland China, *Emerg Infect Dis* **21**:2254–2255, 2015.
10. Lorsirigool A, Saeng-Chuto K, Temeeyasen G, Madapong A, Tripipat T, Wegner M, Tuntituvanont A, Intrakamhaeng M, Nilubol D, The first detection and full-length genome sequence of porcine deltacoronavirus isolated in Lao PDR, *Arch Virol* **161**:2909–2911, 2016.
11. Hsu TH, Liu HP, Chin CY, Wang C, Zhu WZ, Wu BL, Chang YC, Detection, sequence analysis, and antibody prevalence of porcine deltacoronavirus in Taiwan, *Arch Virol* **163**:3113–3117, 2018.
12. Jung K, Hu H, Eyerly B, Lu Z, Chepngeno J, Saif LJ, Pathogenicity of 2 porcine deltacoronavirus strains in gnotobiotic pigs, *Emerg Infect Dis* **21**:650–654, 2015.
13. Chen Q, Gauger P, Stafne M, Thomas J, Arruda P, Burrough E, Madson D, Brodie J, Magstadt D, Derscheid R, Welch M, Zhang J, Pathogenicity and pathogenesis of a United States porcine deltacoronavirus cell culture isolate in 5-day-old neonatal piglets, *Virology* **482**:51–59, 2015.
14. Lin CN, Chung WB, Chang SW, Wen CC, Liu H, Chien CH, Chiou MT, US-like strain of porcine epidemic diarrhea virus outbreaks in Taiwan, 2013–2014, *J Vet Med Sci* **76**:1297–1299, 2014.
15. Lin JD, Lin CF, Chung WB, Chiou MT, Lin CN, Impact of mated female nonproductive days in breeding herd after porcine epidemic diarrhea virus outbreak, *PLoS One* **11**:e0147316, 2016.
16. Sinha A, Gauger P, Zhang J, Yoon KJ, Harmon K, PCR-based retrospective evaluation of diagnostic samples for emergence of porcine deltacoronavirus in US swine, *Vet Microbiol* **179**:296–298, 2015.
17. Marthaler D, Raymond L, Jiang Y, Collins J, Rossow K, Rovira A, Rapid detection, complete genome sequencing, and phylogenetic analysis of porcine deltacoronavirus, *Emerg Infect Dis* **20**:1347–1350, 2014.
18. Ma Y, Zhang Y, Liang X, Lou F, Oglesbee M, Krakowka S, Li J, Origin, evolution, and virulence of porcine deltacoronaviruses in the United States, *MBio* **6**:e00064, 2015.
19. Vester B, Wengel J, LNA (locked nucleic acid): High-affinity targeting of complementary RNA and DNA, *Biochemistry* **43**:13233–13241, 2004.
20. Braasch DA, Corey DR, Locked nucleic acid (LNA): Fine-tuning the recognition of DNA and RNA, *Chem Biol* **8**:1–7, 2001.
21. Jung K, Wang Q, Scheuer KA, Lu Z, Zhang Y, Saif LJ, Pathology of US porcine epidemic diarrhea virus strain PC21A in gnotobiotic pigs, *Emerg Infect Dis* **20**:662–665, 2014.
22. Bjustrom-Kraft J, Woodard K, Gimenez-Lirola L, Rotolo M, Wang C, Sun Y, Lasley P, Zhang J, Baum D, Gauger P, Main R, Zimmerman J, Porcine epidemic diarrhea virus (PEDV) detection and antibody response in commercial growing pigs, *BMC Vet Res* **12**:99, 2016.
23. Jung K, Hu H, Saif LJ, Porcine deltacoronavirus infection: Etiology, cell culture for virus isolation and propagation, molecular epidemiology and pathogenesis, *Virus Res* **226**:50–59, 2016.
24. Kim Y, Yang M, Goyal SM, Cheeran MC, Torremorell M, Evaluation of biosecurity measures to prevent indirect transmission of porcine epidemic diarrhea virus, *BMC Vet Res* **13**:89, 2017.

Taiwan Vet J Downloaded from www.worldscientific.com
by Fong Kiaw Wong on 11/01/21. Re-use and distribution is strictly not permitted, except for Open Access articles.
