# Supplementary material for: Phylodynamic analysis and spike protein mutations in porcine deltacoronavirus with a new variant introduction in Taiwan
Source: Virus Evol. 2021 Nov 24;7(2):veab096. doi: 10.1093/ve/veab096 (PMC8634457; doi:10.1093/ve/veab096)
Supplement: veab096_Supp [file veab096_supp.zip › Supplementary Tables.docx]

**Supplementary Tables**

**Table 1. Primers for the full-length spike gene sequencing.**

| **Primer name** | **Sequences (5’-3’)** | **Nucleotide position** | **Reference** |
| --- | --- | --- | --- |
| PDCoV-SF1 | TATTATCTCGGCTCGTGAG | 19241-20070 | Liu et al., 2018 |
| PDCoV-SR1 | AGTGTTATGAGTGTATCGG |  | Liu et al., 2018 |
| PDCoV-SF2 | CGGTTAACTATGTTAGGTTGTATCG | 19980-20831 | This study |
| PDCoV-SR2 | ATGTGATAGCACCGACAACG |  | This study |
| PDCoV-SF3 | AACAGGTGAGCTTTATGC | 20713-21529 | This study |
| PDCoV-SR3 | AGAGCCAGTATACATTGCC |  | Liu et al., 2018 |
| PDCoV-SF4 | TCTAGAGACATGGCCATCG | 21422-22228 | Liu et al., 20178 |
| PDCoV-SR4 | CTGGTAGAGTATAAGTTGAGGC |  | This study |
| PDCoV-SF5 | TTTTCATGCATGCAGTGCT | 22113-22874 | Liu et al., 2018 |
| PDCoV-SR5 | CCAATGCAGATGACAACTAGT |  | This study |

*Nucleotide positions are based on the reference porcine deltacoronavirus strain HKU15-44 (GenBank Accession No. JQ065042).

**Table 2. PDCoV reference strains used in the recombination analysis**

| **Groups of names** | **PDCoV reference strains (GenBank Accession Number)** |
| --- | --- |
| PDCoV Chinese lineage 1.1 | CHJXNI2 (KR131621), CH-XS (MK040452), CHN-JS (MN249445), CC-HNZK-02 (MK248485), HeN (MN942260) |
| PDCoV Chinese lineage 1.2 | AH2018641 (MN058056), SD-07 (MN173807), Sichuan-2019 (MK993519), CH-HG (MF095123), SCNC201705 (MH051845) |
| PDCoV Others | CH-XJYN (MN064712), CH-Jiangsu (KY513725), CH-01 (KY513725), AH-2004 (KP757890), CHN-QH (MF642325) |
| PDCoV Prototype lineage | HKU15-44 (JQ065042), CHN-HB (KP757891) |
| PDCoV Southeast Asia lineage | HaNoi6 (KX834351), P1 12 ST1 0213 (KX361343), S5016 (KU051650), TT 1115 (KU984334),  P1 12 BTL 0115 (KX118627), |
| PDCoV USA lineage | Minnesota (KR265853), SL5 (KR060083), HKD (LC260043), OAXUI1253CMPR (MK478383), Minnesota292 (KR265864) |
